# Electrocardiographic characteristics of newborns with ventricular septal defects: a Copenhagen Baby Heart Study

**DOI:** 10.1007/s00431-023-05187-7

**Published:** 2023-09-11

**Authors:** Christian Pihl, Maria Munk Pærregaard, Anne-Sophie Sillesen, Ruth Ottilia B Vøgg, Adrian Pietersen, Anna Axelsson Raja, Kasper Karmark Iversen, Henning Bundgaard, Alex Hørby Christensen

**Affiliations:** 1 Department of Cardiology, Copenhagen University Hospital - Herlev-Gentofte Hospital, Borgmester Ib Juuls Vej 1, 2730 Herlev, Copenhagen, Denmark; 2 Department of Cardiology, The Heart Centre, Copenhagen University Hospital - Rigshospitalet, Copenhagen, Denmark; 3 Department of Clinical Medicine, University of Copenhagen, Copenhagen, Denmark

**Keywords:** Ventricular septal defect, Copenhagen Baby Heart Study, CBHS, Congenital heart defect

## Abstract

Ventricular septal defects (VSD) represent the most common congenital heart defect in newborns. We assessed the electrocardiographic characteristics of newborns with VSDs in a general population sample. The Copenhagen Baby Heart Study is a prospective population–based cohort study offering cardiac evaluation of newborns. Echocardiograms and electrocardiograms were obtained within 30 days after birth and systematically analysed. A VSD was identified in 530 newborns (mean age 11 ± 7 days, 42% boys). Newborns with VSDs had a more left-shifted *QRS* axis (116 ± 34 vs. 120 ± 3°, *p* = 0.02), and a higher *S*-wave amplitude in V1 (721 ± 584 vs. 636 ± 549 µV, *p* = 0.001) than controls. The largest differences were found in newborns with large or perimembraneous VSDs with a higher frequency of left axis deviation, higher *S*-wave amplitudes in V1, and higher *R*- and *S*-wave amplitudes in V6 compared with controls. *R*-waves in V1 and V6 were significantly associated to left ventricular mass, whereas *S*-waves in V1 and V6 were dependent on left ventricular end-diastolic diameter on echocardiography.

*  Conclusion*: Newborns with VSDs showed significant differences in *QRS* axis, and *R*- and *S*-wave precordial amplitudes compared to matched controls. Perimembranous and large VSDs had the greatest effect on the neonatal ECG.

**Table:** 

**What is Known:** *• Ventricular septal defects in newborns are prevalent and may affect cardiac function and structure.*	
**What is New:** *• The Copenhagen Baby Heart Study is the largest study including a cohort of unselected newborns undergoing postnatal cardiac examination.* *• We found that newborns with VSD showed significant electrocardiographic differences depending on size and type of VSD compared with healthy newborns.*

**Supplementary Information:**

The online version contains supplementary material available at 10.1007/s00431-023-05187-7.

## Introduction

Congenital heart disease (CHD) is generally reported in ~ 8 per 1000 live births [1], and ventricular septal defects (VSDs) are one the most common types of CHDs [2]. VSDs may result in altered hemodynamics as a consequence of left-to-right shunting, resulting in an increased work load of the left ventricle [3, 4], which eventually may result in left ventricular dilatation, hypertrophy, and systolic dysfunction [5].

The anatomical location of a VSD may further alter the development of the atrioventricular conduction system, leading to conduction abnormalities and arrhythmias [6]. Therefore, early electrocardiographic abnormalities may represent the effects of both hemodynamic and anatomical alterations [3, 6]. Few older studies have explored electrocardiographic patterns, such as the *QRS* axis and *R*- and *S*-wave amplitudes in newborns with VSDs as signs of left and right ventricular loading and have presented criteria hereof, with varying precision [7–9]. However, this topic has not been evaluated in a large general population sample with modern automatized annotation algorithms.

The aim of this study was to describe the electrocardiographic findings in a large cohort of unselected newborns with VSDs from the general population and to investigate the impact of VSD size and anatomical location on electrocardiographic parameters.

## Method

### Study design and population

The Copenhagen Baby Heart Study (CBHS) is a population-based cohort study of the prevalence, spectrum, and prognosis of structural and functional cardiac abnormalities in newborns. The study design has previously been described in detail [10]. In brief, the study was open to all newborns born at one of three largest maternity wards in the greater Copenhagen area from April 1, 2016, to October 31, 2018. The ethnic distribution in the greater Copenhagen area consisted of 76% of Danish origin, 15% non-Western immigrants and their offspring, and 9% Western immigrants and their offspring. All expectant parents were invited to participate and received study information at the routine ultrasound scan in gestational weeks 18–20. ECG, echocardiography, and pulse oximetry were performed within the first 28 days of life. Cases were matched 1:4 with healthy controls by sex, age, and weight at examination.

The study complied with the Declaration of Helsinki, was approved by the Regional Ethics Committee Capital Region of Denmark (H-16001518), and the Data Protection Agency (I-Suite no.: 04546, ID-no. HGH-2016–53). Written informed consent was provided by parents.

### Electrocardiogram

Electrocardiograms were recorded with a MAC 5500 HD system (GE ECG System, Milwaukee, USA) with speed 25 mm/s, sensitivity at 10 mm/µV, sample rate 500 Hz, and bandwidth filter 0.16–150 Hz. We recorded leads I, II, III, aVR, aVL, aVF, V1, and in most cases V6. The ECGs were performed, while the infant was tranquil or sleeping. All tracings were acquired digitally, and intervals, amplitudes, areas, vectors, *QRS* axis, etc., were automatically analysed and stored in the MUSE software (Version 8, GE Healthcare, Milwaukee, USA). *QRS* axis was defined as “adult normal” (+ 1 to + 90°), left axis deviation (LAD; 0 to − 90°), right axis deviation (RAD; + 91 to + 180°), or extreme axis deviation (EAD; + 181 to + 270°) [11, 12].

### Echocardiography

A paediatric echocardiographic examination including 33 pre-defined loops was performed on a Vivid E9 (GE HealthCare System, Horten, Norway), using 6 MHz and 12 MHz probes, in accordance with the American Society of Echocardiography’s guidelines for paediatric echocardiography [13]. Echocardiograms were subsequently analysed using the EchoPAC clinical software (vers. 113, GE HealthCare Systems, Horten, Norway). A VSD was defined as a flow across the interventricular septum in colour Doppler mode in one or more views. Newborns with VSDs as part of complex CHD were excluded. VSDs were classified as either muscular or perimembranous, according to the classification by the International Society for Nomenclature of Pediatric and Congenital Heart Disease [14]. Perimembranous VSDs were further classified as inlet VSDs, when VSDs had extensions to the inlet portion of the right ventricle. Perimembranous VSDs that extended to the outlet septum of the right ventricle were classified as outlet VSDs. The largest visible diameter of the VSD was measured in apical four/five-chamber view, parasternal long axis, or short axis view. The size of VSD was defined as small (VSD < 1/3 of the diameter of the aortic annulus), moderate (VSD ≥ 1/3 but < 2/3 diameter of the aortic annulus), or large (VSD ≥ 2/3 diameter of the aortic annulus), as previously suggested [4, 15]. Newborns with two or more VSDs of the same type were classified as having “multiple VSDs.” Left ventricular mass (LVM) was calculated using Deveraux’s formula [16].

### Statistical analysis

Continuous variables are presented as mean ± standard deviation and categorical variables as absolute numbers with percentages. Fisher’s exact test was used to compare dichotomous variables. Unpaired Student’s *t*-tests and Kruskal–Wallis’ tests were used to compare the differences between groups, when appropriate. Analysis of covariance (ANCOVA) was used to test for effects of covariates on dependent variables in models containing VSD size, VSD type, left ventricular mass, and left ventricular end-diastolic diameter. Linear regression was used to test for trends. A *p*-value < 0.05 was considered statistically significant. All statistical analyses and graphs were made using Rstudio (ver. 1.2.1335, Boston, USA).

## Results

### Study population

For the present study, 530 newborns diagnosed with a VSD and 2120 matched controls were included. Two cases of subarterial VSD were pooled with the perimembranous group. Among cases, the mean age was 11.0 ± 6.6 days (range 0–28 days), and there was a female preponderance (58%, 309 out of 530 newborns). The case and control groups were similar in baseline characteristics (Table 1; all *p* > 0.05). Among the newborns with VSDs, 499 (94%) had a muscular VSD and 31 (6%) had perimembranous VSDs. Small VSDs accounted for 351 (66.2%) cases, moderate-sized VSDs for 105 (19.8%) cases, and large VSDs for 13 (2.5%) cases. Multiple VSDs were found in 61 (11.5%) cases and were all of a muscular type.

**Table 1:** Baseline characteristics of the study population

Characteristics	Newborns with VSD (*n* = 530)	Control group (*n* = 2120)	*p* value
Age, days	11 (7)	11 (7)	0.74
Boys, *n* (%)	221 (42)	884 (42)	1.00
Weight, kg	3.6 (0.5)	3.6 (0.5)	0.59
Height, cm	52 (2)	52 (2)	0.90
Gestational age, days	279 (10)	280 (10)	0.80

Values are presented as mean (SD) or as *n* (%)

### Overall electrocardiographic findings

Newborns with VSDs had a significantly more left-shifted *QRS* axis (116 ± 34 vs. 120 ± 31°, *p* = 0.02). Specifically, we found that newborns with VSDs had significantly less right axis deviation than controls (84 vs. 89%, *p* = 0.02) and that “adult normal” axis was more frequent among newborns with VSDs (12 vs. 7%, *p* = 0.002). We also observed a higher maximum *S*-wave amplitude in V1 (805 ± 568 vs. 714 ± 530 µV, *p* = 0.009) compared with controls.

### Electrocardiographic characteristics by VSD type

#### Different VSD types vs. controls

Newborns with muscular VSDs had a significantly more left-shifted *QRS* axis (117 ± 32 vs. 120 ± 31°, *p* = 0.04) and a higher *S*-wave amplitude in V1 (791 ± 557 vs. 714 ± 530 µV, *p* = 0.006) compared with controls. Newborns with perimembranous VSDs had a significantly higher heart rate (150 ± 22 vs. 142 ± 21 bpm, *p* = 0.03), longer PR interval (106 ± 11 vs. 98 ± 11 ms, *p* < 0.001), lower frequency of right axis deviation (65 vs. 89%, *p* = 0.03), and higher S-wave amplitudes in both V1 (1037 ± 708 vs. 714 ± 530 µV, *p* < 0.001) and V6 (939 ± 726 vs. 705 ± 396 µV, *p* = 0.01) compared with controls. Electrocardiographic measurements by VSD type are summarized in Table 2.

**Table 2:** Electrocardiographic findings in newborns with muscular and perimembranous VSDs

Variable	Muscular (*n* = 499)	*p* value	Perimenbranous, inlet (*n* = 26)	*p* value	Perimenbranous, outlet (*n* = 5)	*p* value	Control group (*n* = 2120)
Electrocardiographic findings							
Heart rate, bpm	142 (21)	0.96	150 (23)	**0.04**	148 (16)	0.52	142 (22)
PR-interval, ms	98 (11)	0.37	105 (10)	** < 0.01**	110 (15)	**0.03**	98 (11)
QRS duration, ms	54 (6)	0.17	55 (7)	0.90	56 (6)	0.72	55 (6)
QRS axis, degrees	117 (32)	**0.04**	96 (44)	** < 0.01**	198 (90)	** < 0.01**	120 (31)
”Adult normal” axis, *n* (%)	41 (10.8%)	**0.008**	5 (25)	**0.01**	0 (0.00)	1.00	103 (6.7%)
Right axis deviation, *n* (%)	322 (85%)	**0.076**	14 (70)	**0.03**	1 (33)	**0.04**	1369 (88.7%)
Left axis deviation, *n* (%)	1 (0.3%)	0.82	1 (5)	0.25	0 (0.0)	1.00	8 (0.5%)
Extreme axis deviation, *n* (%)	14 (3.7%)	0.81	0 (0.0)	0.72	2 (67)	** < 0.01**	64 (4.1%)
Max R amplitude in V1, µV	1171 (565)	0.89	1104 (577)	0.52	1487 (1129)	0.21	1175 (553)
Max S amplitude in V1, µV	791 (557)	**0.006**	882 (616)	0.12	1967 (500)	** < 0.01**	714 (530)
Max R amplitude in V6, µV	993 (480)	0.14	1135 (561)	0.09	297 (0.0)	0.10	952 (447)
Max S amplitude in V6, µV	725 (441)	0.43	933 (748)	**0.02**	1040 (0.0)	0.29	705 (396)
QT interval, ms	277 (25)	0.76	275 (23)	0.82	272 (21)	0.72	277 (26)
QTc (Bazett), ms	420 (25)	0.67	423 (25)	0.57	418 (15)	0.89	420 (25)
Echocardiographic findings							
Fractional shortening, %	34.2(4.7)	** < 0.01**	34.4 (3.6)	**0.04**	32.0 (3.2)	0.74	32.6 (4.2)
Ejection fraction, %	65.9 (6.2)	** < 0.001**	66.2 (4.7)	0.05	63.0 (5.0)	0.76	63.9 (6.0)
Left ventricular end-diastolic diameter, mm	20.5 (1.9)	** < 0.001**	21.1 (2.3)	** < 0.01**	20.5 (2.5)	0.45	19.9 (1.8)
Left ventricular end-systolic diameter, mm	13.5 (1.5)	0.30	13.8 (1.4)	0.13	14.0 (2.2)	0.37	13.4 (1.4)
Interventricular septum, mm	2.2 (0.4)	** < 0.001**	2.4 (0.4)	0.08	2.4 (0.3)	0.64	2.5 (0.5)
Left ventricular posterior wall, mm	1.8 (0.4)	** < 0.001**	1.8 (0.4)	**0.04**	2.0 (0.4)	0.69	2.1 (0.7)
Left ventricular mass, g	5.7 (1.4)	** < 0.001**	6.3 (1.6)	0.97	6.1 (1.3)	0.70	6.4 (1.4)

Values are presented as mean (SD) or as *n* (%). All *p*-values are comparisons with the control group. Significant *p*-values are marked with bold

#### Muscular vs. perimembranous VSD

Newborns with muscular VSDs had a lower heart rate (142 ± 21 vs. 150 ± 22 bpm, *p* = 0.033), a shorter PR interval (98 ± 11vs. 106 ± 11 ms, *p* = 0.001), a higher proportion of right axis deviation (85 vs. 65%, *p* = 0.025), and lower *S*-wave amplitude in V1 (791 ± 557 vs. 1037 ± 708 µV, *p* = 0.026) compared with newborns with perimembranous VSDs.

### Electrocardiographic characteristics by VSD size

#### VSD size vs. controls

**Fig. 1:**
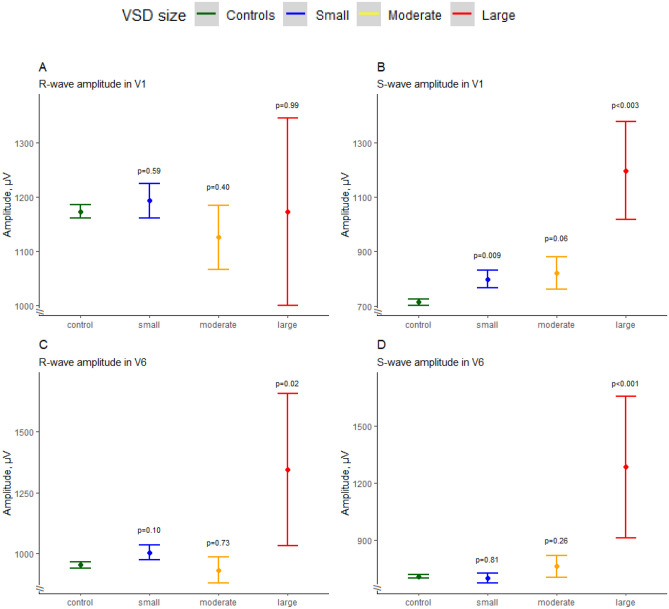
**A** Maximum *R*-wave amplitude in lead V1, grouped by VSD size. **B** Maximal *S*-wave amplitude in lead V1, grouped by VSD size. **C** Maximal *R*-wave amplitude in V6 grouped by VSD size. **D** Maximal *S*-wave amplitude in V6 grouped by VSD size. Dots represent mean values, and bars represent ± 95% confidence intervals

Newborns with small VSDs showed a higher maximum *S*-wave amplitude in V1 (798 ± 580 vs. 714 ± 530 µV, *p* = 0.009) compared with controls whereas newborns with moderate VSDs had higher proportion of “adult normal” axis (13.6 vs. 6.7%, *p* = 0.03) compared with controls (Fig. 1). Newborns with large VSDs had a significantly longer PR interval (105 ± 14 vs. 98 ± 11 ms, *p* = 0.04), less right axis deviation (56 vs. 89%, *p* = 0.01), higher *S*-wave amplitude in V1 (1196 ± 596 vs. 714 ± 530 µV, *p* = 0.003) S-V6 (1285 ± 985 vs. 705 ± 396 µV, *p* < 0.001), and *R*-wave amplitude in V6 (1345 ± 822 vs. 952 ± 447 µV, *p* = 0.02) compared with controls. Echocardiographic findings subgrouped by VSD size are summarized in Supplementary Table 1.

#### Small/moderate vs. large VSD

Newborns with large VSDs were more likely to have an *QRS* axis within the interval defined as with left axis deviation (11.1 vs. 0.3%, *p* = 0.040), extreme axis deviation (22.2 vs. 3.4%, *p* = 0.044), and less right axis deviation (55.6 vs. 85.8%, *p* = 0.041) compared with newborns with small/moderate VSDs. Newborns with large VSDs furthermore had a higher *S*-wave amplitude in V1 (1196 ± 596 vs. 803 ± 578 µV, *p* = 0.027) and *S*-wave amplitude in V6 (1285 ± 985 vs. 712 ± 425 µV, *p* = 0.001) compared with newborns with small/moderate VSDs.

#### Muscular VSDs by size vs controls

Newborns with small muscular VSDs had significantly larger *S*-wave amplitudes in V1 (795 ± 580 vs 714 ± 530 µV, *p* < 0.01) compared to newborns without VSDs but had otherwise similar ECGs.

Newborns with large muscular VSDs had a significantly larger S-wave amplitude in V6 (1313.0 ± 1312 vs. 705 ± 396 µV, *p* = 0.03) and a longer QTcB interval (450 ± 48 vs. 420 ± 25 ms, *p* = 0.04) compared with newborns without VSDs.

### Perimembranous VSDs vs controls

Newborns with small perimembranous VSDs had comparable ECGs with newborns without VSDs.

Newborns with large perimembranous outlet VSDs had a significantly larger PR interval (121 ± 10 vs. 98 ± 11°, *p* < 0.01), a significantly higher proportion of extreme axis deviation (2 (100%) vs. 64 (4.1%), *p* < 0.01) and a larger *S*-wave amplitude in V1 (2155 ± 438 vs. 714 ± 530 µV, *p* < 0.01) compared with newborns without VSDs.

Newborns with large perimembranous inlet VSDs had significantly more left-shifted *QRS* axis (57 ± 72 vs 120 ± 31°, *p* < 0.01) and a higher proportion of left axis deviation (1 (25%) vs 8 (0.5%), *p* < 0.01), and significantly higher *R*- and *S*-wave amplitudes in V6 (1711.0 ± 768 vs. 952 ± 447 µV, *p* < 0.01 and 1332 ± 1159 vs 705 ± 396 µV, *p* < 0.01). Electrocardiographic findings are summarized in Table 3.

**Table 3:** Electrocardiographic findings in newborns with VSDs, divided by size and type of VSD, compared with newborns without VSDs

Variable	**Muscular small (*n* = 343)**	***p* value**	**Muscular large (*n* = 3)**	***p* value**	**Perimembranous small (*n* = 8)**	***p* value**	**Perimembraous outlet large (*n* = 3)**	***p* value**	**Perimembranous Inlet large (*n* = 7)**	***p* value**	**Control group (*n* = 2120)**
Electrocardiographic findings											
Heart rate, bpm	142 (22)	0.90	146 (35)	0.71	144 (25)	0.76	156 (12)	0.27	154 (29)	0.13	142 (22)
PR-interval, ms	98 (11)	0.68	88 (9)	0.19	103 (11)	0.23	121 (10)	** < 0.01**	105 (10)	0.12	98 (11)
QRS duration, ms	55 (6)	0.71	56 (6)	0.70	52 (6)	0.23	59 (5)	0.26	57 (7)	0.28	55 (6)
QRS axis, degrees	116 (31)	0.05	112 (19)	0.66	118 (26)	0.84	250 (18)	** < 0.01**	57 (72)	** < 0.01**	120 (31)
”Adult normal” axis, *n* (%)	11 (3)	0.23	0 (0.0)	1.00	0 (0.0)	1.00	0 (0.0)	1.00	1 (25)	0.64	103 (6.7%)
Right axis deviation, *n* (%)	230 (87)	0.35	3 (100)	1.00	6 (100)	0.82	0 (0.0)	< 0.01	2 (50)	0.10	1369 (88.7%)
Left axis deviation, *n* (%)	1 (0.4)	1.00	0 (0.0)	1.00	0 (0.0)	1.00	0 (0.0)	1.00	1 (25)	** < 0.01**	8 (0.5%)
Extreme axis deviation, *n* (%)	9 (3.4)	0.68	0 (0.0)	1.00	0 (0.0)	1.00	2 (100)	** < 0.01**	0 (0.0)	1.00	64 (4.1%)
Max R Amplitude in V1, µV	1190 (586)	0.65	1493 (785)	0.32	1315 (605)	0.48	1233 (604)	0.86	1010 (601)	0.43	1175 (553)
Max S Amplitude in V1, µV	795 (580)	**0.01**	831 (412)	0.70	941 (628)	0.23	2155 (438)	** < 0.01**	1059 (367)	0.11	714 (530)
Max R Amplitude in V6, µV	1006 (482)	0.09	1137 (752)	0.56	885 (310)	0.76	297 (0.0)	0.10	1711 (768)	** < 0.01**	952 (447)
Max S Amplitude in V6, µV	700 (415)	0.85	1313 (1312)	**0.03**	631 (373)	0.71	1040 (0.0)	0.29	1332 (1159)	** < 0.01**	705 (396)
QT interval, ms	278 (26)	0.54	291 (10)	0.36	276 (31)	0.94	267 (18)	0.60	283 (6)	0.67	277 (26)
QTc (Bazett), ms	421 (24)	0.51	450 (48)	**0.04**	422 (18)	0.84	421 (16)	0.95	415 (17)	0.73	420 (25)
Echocardiographic findings											
Fractional shortening, %	34.1 (4.7)	** < 0.01**	35.9 (2.3)	0.18	34.1 (2.4)	0.33	30.4 (3.2)	0.35	35.9 (3.3)	**0.04**	32.6 (4.2)
Ejection fraction, %	65.7 (6.3)	** < 0.01**	68.4 (3.4)	0.19	66.0 (3.4)	0.32	60.4 (4.9)	0.33	68.0 (4.1)	0.07	63.9 (6.0)
Left ventricular end-diastolic diameter, mm	20.4 (1.8)	** < 0.01**	20.6 (2.9)	0.49	20.5 (1.3)	0.34	22.0 (1.7)	**0.04**	22.4 (3.4)	** < 0.01**	19.9 (1.8)
Left ventricular end-systolic diameter, mm	13.5 (1.4)	0.44	13.2 (2.3)	0.83	13.5 (1.3)	0.80	15.3 (1.4)	**0.02**	14.3 (2.0)	0.08	13.4 (1.4)
Interventricular septum, mm	2.2 (0.4)	** < 0.01**	2.1 (0.6)	0.10	2.4 (0.2)	0.58	2.2 (0.0)	0.29	2.4 (0.3)	0.35	2.5 (0.5)
Left ventricular posterior wall, mm	1.8 (0.4)	** < 0.01**	1.9 (0.3)	0.52	1.7 (0.2)	0.07	1.9 (0.4)	0.56	2.0 (0.4)	0.78	2.1 (0.7)
Left ventricular mass, g	5.7 (1.4)	** < 0.01**	5.9 (2.2)	0.53	5.9 (0.7)	0.31	6.5 (2.0)	0.90	7.4 (2.2)	**0.05**	6.4 (1.4)

Values are presented as mean (SD) or as *n* (%). All *p*-values are comparisons with the control group. Significant *p*-values are marked with bold

### Echocardiographic findings and their association with ECG parameters

Newborns with VSDs had higher ejection fractions (65.9 ± 6.1 vs. 63.9 ± 6.0%, *p* < 0.001), larger left ventricular end-diastolic diameter (LVIDd) (20.5 ± 1.9 vs. 19.9 ± 1.8 mm, *p* < 0.001), thinner interventricular septum (2.2 ± 0.4 vs. 2.5 ± 0.5 mm, *p* < 0.001) and left ventricular posterior wall (1.8 ± 0.4 vs. 2.1 ± 0.7 mm, *p* < 0.001), and lower left ventricular muscle mass (5.7 ± 1.4 vs. 6.4 ± 1.4 g, *p* < 0.001) compared with controls.

**Fig. 2:**
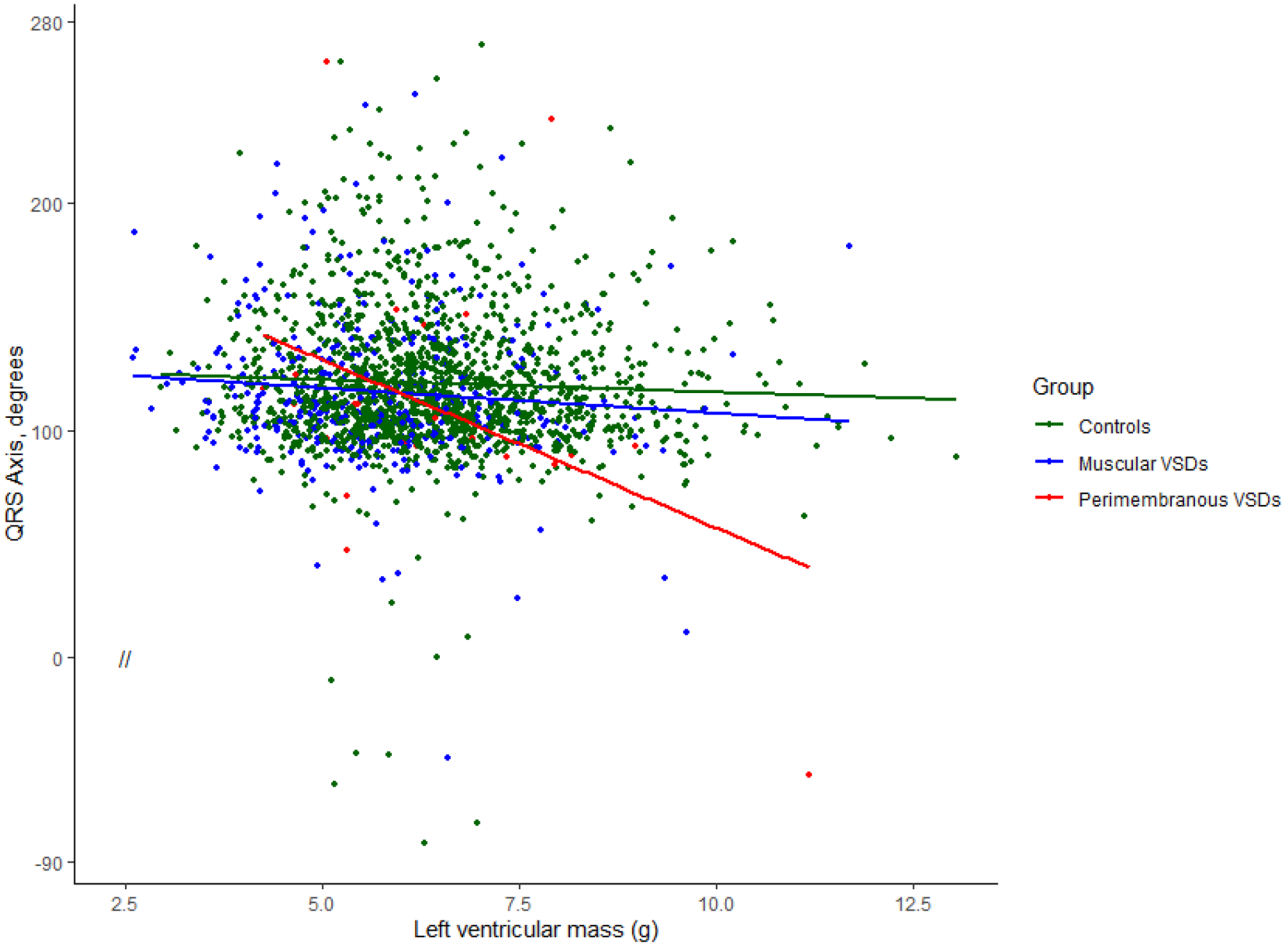
Linear regression of the QRS axis as a function of left ventricular mass (in grams), grouped by VSD type, and compared with controls. Perimembranous VSDs (red line) had significant influence on the effect of left ventricular mass on the QRS axis compared with controls (green line) and muscular VSDs (blue line; *p* = 0.0011)

We investigated the association between LVM and the *QRS* axis subgrouped by VSD type and size. We found that LVM (*p* = 0.012) and type of VSD (*p* = 0.011) were significantly correlated to the *QRS* axis towards a leftward-shifted axis for newborns with VSDs. Furthermore, the effect of LVM on the *QRS* axis was significantly influenced by perimembranous type VSDs (*p* = 0.001) (Fig. 2).

**Fig. 3:**
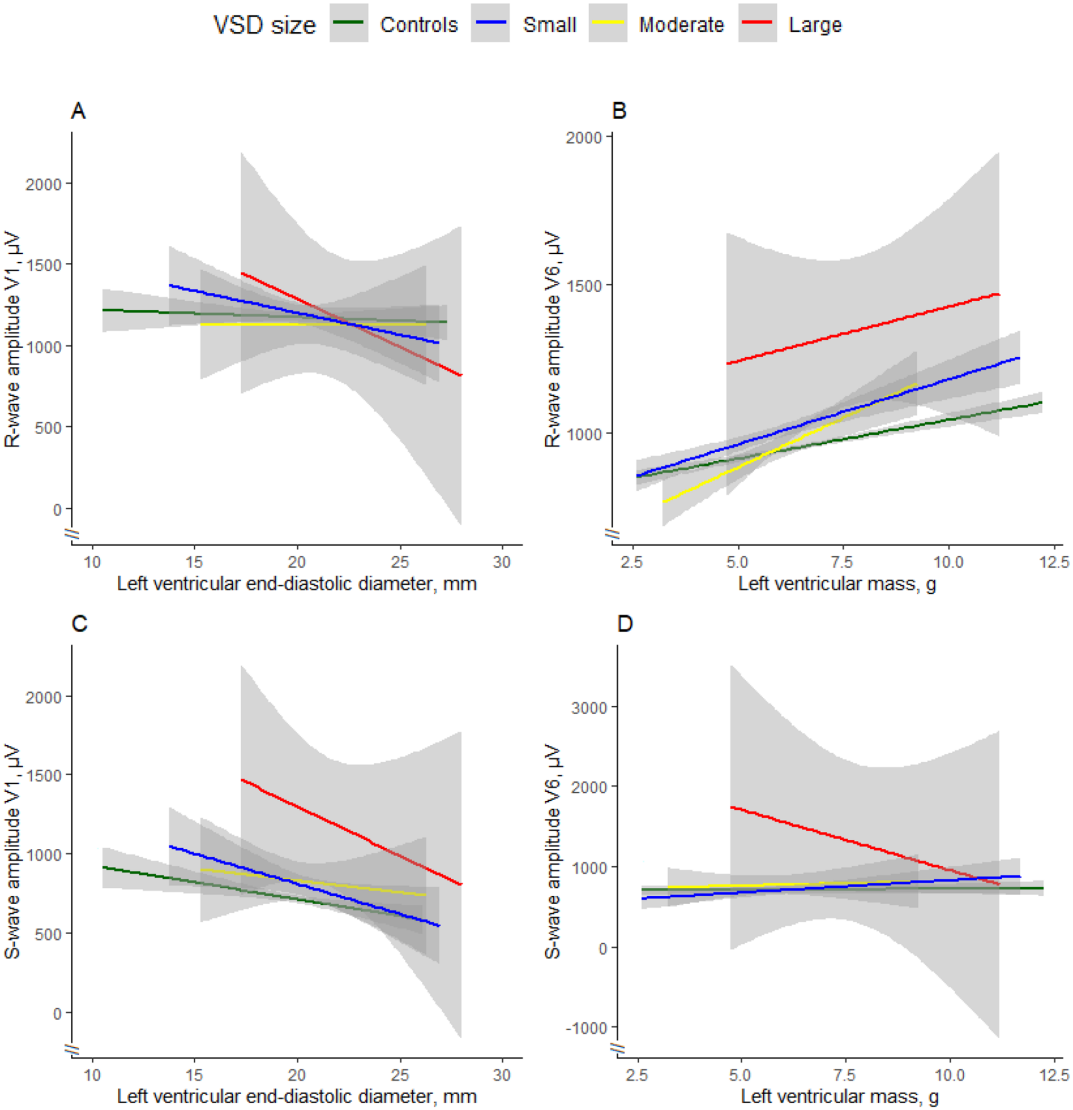
**A** *R*-wave amplitude in V1 as a function of left ventricular mass (LVM, in grams), grouped by VSD size. Though LVM was found to be significantly associated with *R*-wave amplitude in V1, when subgrouping by VSD size, no statistical significance for any VSD size was found. **B** *R*-wave amplitude in V6 as a function of left ventricular mass grouped by VSD size. When controlling for the effect of LVM, the V6 *R*-wave amplitude was influenced by the size of the VSD, and was significantly increased for large VSDs (*p* = 0.04). **C** *S*-wave amplitude in V1 as a function of left ventricular end-diastolic diameter (LVIDd), grouped by VSD size. When controlling for the effect of LVIDd, the V1 *S*-wave amplitude was influenced by the size of the VSD, and was significantly increased for small (*p* = 0.0056), moderate (*p* = 0.035), and large VSDs (*p* < 0.001) compared with controls. **D** *S*-wave amplitude in V6 as a function of LVIDd, grouped by VSD size. When controlling for the effect of LVIDd, the *S*-wave amplitude in V6 was influenced by the size of the VSD, and was significantly increased for large VSDs (*p* < 0.001) compared with controls. Shaded grey area represent 95% confidence interval

We analysed the maximum *R*- and *S*-wave amplitudes in V1 and V6 as functions of LVM and LVIDd by VSD size. The *R*-wave amplitudes in V1 and V6 were found to be most significantly associated with LVM (*p* = 0.033 and *p* < 0.001 respectively) (Fig. 3A + C). *S*-wave amplitudes in V1 and V6 was found to be most significantly associated with LVIDd (*p* = 0.0034 and *p* = 0.01 respectively) (Fig. 3B + D)*.* Taken together, these results suggest that perimembranous VSDs affect the *QRS* axis not solely through ventricular remodelling, as is indicated by the *R*- and *S*-wave amplitudes*.*

## Discussion

In the present study of unselected newborns with VSDs from a general population sample, we found most noticeable electrocardiographic differences in the *QRS* axis and precordial *R*- and *S*-wave amplitudes compared with matched controls. The electrocardiographic differences were dependent on VSD size and location, with large and perimembranous VSDs having the most pronounced effect. We furthermore found associations between precordial amplitudes and left ventricular measurements on echocardiography.

We found a significant, but relatively small, difference in the *QRS* axis with a more left-shifted *QRS* axis and an increased *S*-wave amplitude in V1 in newborns with VSDs compared with matched controls. The *QRS* axis in newborns has previously been associated with CHD in children, where up to 66% of newborns with LAD were found to have a structural heart disease [11, 17]. However, a recent study reported a low frequency of LAD in a large unselected general population cohort of newborns with structurally normal hearts [18]. However, we only found LAD in 0.5% of the newborns with VSDs, which was similar to the percentage found in the matched controls.

Investigation of electrocardiographic parameters among newborns with perimembranous VSDs revealed a prolonged PR interval, a more left-shifted *QRS* axis with a higher frequency of LAD and lower frequency of RAD, and higher *S*-wave amplitude in V6. These findings may be explained by subtle anatomical abnormalities e.g. in Koch’s triangle resulting in increased conduction time through the right atrium [19]. Sodi-Pallares et al. noted that the majority of their patients with VSDs had a prolonged *P*-wave duration and 12% were found to have a first-degree atrioventricular block [9]. Vince et al. reported similar results, although with a lower rate of *P*-wave prolongation (30%) and PR interval > 180 ms (6%) [7]. Previous studies have also found that the anterior VSDs had little or no relation to the conduction system, whereas posterior VSDs and VSDs closer to the AV-node, changed the course of the of the conduction system and thereby increased conduction times [6, 19]. We found significant differences in *QRS* axis in both large inlet and outlet perimembranous VSDs. This may also be attributed to anatomical variations. Titus et al. found that subjects with VSDs placed posterior and inferior to the crista supraventricularis were found to have a posterior displacement of the AV node and bundle branch variations from the normal.

Newborns with large VSDs had longer PR interval, higher *S*-waves in V1 and V6, and higher *R*-waves in V6. Previous studies have suggested that *R*- and *S*-wave abnormalities may be signs of left ventricular strain associated with VSDs [3, 7, 8]. Sodi-Pallares et al. found that tall *R*-waves in V1 were strong indicators of right ventricular overload in children with VSDs [9]. In line with these findings, Vince et al. reported that tall *R*-waves in V6 > 200 µV, *S* waves in V1 ≥ 250 µV, or *QRS* axis < 90° were the most important ECG criteria for elevated pulmonary vascular resistance in children with VSDs (age 4 months to 4 years) and left ventricular or biventricular loading [7]. However, these older studies included children with a much wider age spectrum, and with variable degrees of pulmonary hypertension, and may therefore not be directly comparable.

We found that *R*-waves in V1 and V6 were associated with the LVM, whereas *S*-waves in V1 and V6 were associated with left ventricular end-diastolic diameter. Newborns with small and moderate VSDs had thinner interventricular septums and posterior walls as observed on echocardiography, but a larger end-diastolic left ventricular diameter, which resulted in a small increase of the *R*- and *S*-wave amplitudes on the ECG. This likely reflect left ventricular dilation due to the volume overload, but without myocardial hypertrophy. Newborns with large VSDs were found to have the highest LVM and significantly larger left ventricular end-diastolic diameters, and consequently taller *R*-waves in V6 and the reciprocal *S*-waves in V1. Similarly, newborns with perimembranous VSDs, who on average had large VSDs and presumably higher shunt ratio, had larger left ventricular end-diastolic diameters and higher *S*-waves in V6 compared to controls.

The ECG is an important screening tool for clinicians and is frequently used in the evaluation of newborns, as it is inexpensive, noninvasive, and widely available. Certain findings, such as signs of right or left ventricular hypertrophy, unusual for the age of the newborn, can be suggestive of underlying pathology [20]. It has previously been suggested that CHD may be detected by early ECG screening [5, 21]. In the normal heart, the right ventricle is primarily depicted on the ECG as the amplitudes of the *R*- and *S*-waves in the right precordial leads. Over time, the V1 amplitudes regress and prominent *R*-waves shift towards the left precordial leads [22]. Though we identified certain electrocardiographic signs of VSD that consistently changed either by type or size of VSD, we also found newborns with VSDs to have significant overlap in ECG parameters with healthy controls. It therefore seems unlikely that sensitive detection of congenital VSDs, one of the most common CHDs in newborns, would be feasible with routine ECG screening. In concordance, a study by Danford et al. found that the addition of an ECG did not significantly enhance the diagnostic accuracy for VSDs of all sizes and the ECG is unlikely to change the clinical course in the majority of cases. However, our findings do support that early, smaller variation in ECG parameters do exist between children with and without VSDs suggestive of shared etiologic factors between VSD and the conduction system and that ECG findings may not solely be secondary to hemodynamic effects. Worth noting is that our study population mainly included asymptomatic newborns from the general population and that symptomatic newborns with large VSDs likely exhibit more pronounced ECG findings.

This study is not without limitations. Firstly, the ECGs were recorded with eight leads due to considerations of participant discomfort. Secondly, we obtained only one ECG per newborn and therefore cannot describe ECG changes over time in the same individual. Thirdly, our study population consisted of a small number of newborns with large VSDs. Lastly, the newborns in this study were all asymptomatic at the time of examination and may not be representative of symptomatic newborns referred for evaluation in a clinical setting.

## Conclusion

In conclusion, systematic ECG analysis of unselected newborns with VSDs showed abnormalities in the *QRS* axis and *R*- and *S*-wave precordial amplitudes. These changes in the ECG were found to be significantly associated with type and size of VSDs and the left ventricular mass and diameter measured by echocardiography. Perimembranous VSDs and large VSDs had the greatest effect on the neonatal ECG.

### Supplementary Information

Below is the link to the electronic supplementary material.

Supplementary file1 (DOCX 20 KB)

## Data Availability

The datasets generated during and/or analysed during the current study are available from the corresponding author on reasonable request.
