# Supplementary material for: Electrocardiographic characteristics of newborns with ventricular septal defects: a Copenhagen Baby Heart Study
Source: Eur J Pediatr. 2023 Sep 11;182(11):5149–58. doi: 10.1007/s00431-023-05187-7 (PMC10640423; doi:10.1007/s00431-023-05187-7)
Supplement: Supplementary file 1 — Supplementary file1 (DOCX 20 KB) [file 431_2023_5187_MOESM1_ESM.docx]

Supplementary

Table 1

| Table 1: Electrocardiographic findings in newborns with VSD, subgrouped by VSD size. | | | | | | | | | |  |
| --- | --- | --- | --- | --- | --- | --- | --- | --- | --- | --- |
| Variable | Small  (n=351) | P value | Moderate  (n=105) | P value | Large  (n=13) | P value | Control group  (n=2,120) | | | |
| Electrocardiographic findings |  |  |  |  |  |  |  | | | |
| - Heart rate, bpm | 142 (22) | 0.87 | 143 (19) | 0.52 | 153 (26) | 0.07 | 142 (22) | | | |
| - PR-interval, ms | 98 (11) | 0.57 | 100 (9) | 0.14 | 105 (14) | **0.04** | 98 (11) | | | |
| - QRS duration, ms | 54 (6) | 0.59 | 55 (6) | 0.82 | 57 (6) | 0.13 | 55 (6) | | | |
| - QRS axis, degrees | 116 (31) | 0.052 | 118 (31) | 0.47 | 118 (91) | 0.83 | 120 (31) | | | |
| - ”Adult normal” axis, n (%) | 26 (9.6%) | 0.11 | 11 (13.6%) | **0.03** | 1 (11.1%) | 1.0 | 103 (6.7%) | | | |
| - Right axis deviation, n (%) | 236 (86.8%) | 0.42 | 67 (82.7%) | 0.15 | 5 (55.6%) | **0.01** | 1,369 (88.7%) | | | |
| - Left axis deviation, n (%) | 1 (0.4%) | 1.0 | 0 (0%) | 1.0 | 1 (11.1%) | **0.049** | 8 (0.5%) | | | |
| - Extreme axis deviation, n (%) | 9 (3.3%) | 0.63 | 3 (3.7%) | 1.0 | 2 (22.2%) | 0.064 | 64 (4.1%) | | | |
| - Max R Amplitude in V1, µV | 1,193 (586) | 0.59 | 1,126 (571) | 0.40 | 1,173 (622) | 0.99 | 1,175 (553) | | | |
| - Max S Amplitude in V1, µV | 798 (580) | **0.009** | 821 (571) | 0.06 | 1,196 (596) | **0.003** | 714 (530) | | | |
| - Max R Amplitude in V6, µV | 1,004 (479) | 0.10 | 933 (450) | 0.73 | 1,345 (822) | **0.02** | 952 (447) | | | |
| - Max S Amplitude in V6, µV | 699 (414) | 0.81 | 761 (463) | 0.26 | 1,285 (985) | **<0.001** | 705 (396) | | | |
| - QT interval, ms | 278 (26) | 0.55 | 276 (23) | 0.64 | 282 (14) | 0.57 | 277 (26) | | | |
| - QTc (Bazett), ms | 421 (24) | 0.50 | 421 (27) | 0.72 | 430 (32) | 0.27 | 420 (25) | | | |
| Echocardiographic findings |  |  |  |  |  |  |  | | | |
| - Fractional shortening, % | 34.1 (4.6) | **<0.001** | 34.3 (4.6) | **<0.001** | 34.6 (3.7) | 0.09 | 32.6 (4.2) | | | |
| - Ejection fraction | 65.7 (6.2) | **<0.001** | 66.0 (6.0) | **<0.001** | 66.3 (5.1) | 0.14 | 63.9 (6.0) | |  |  |
| - Left ventricular end-diastolic diameter, mm | 20.5 (1.8) | **<0.001** | 20.8 (2.0) | **<0.001** | 21.9 (2.9) | **<0.001** | 19.9 (1.8) | |  |  |
| - Left ventricular end-systolic diameter, mm | 13.5 (1.4) | 0.40 | 13.6 (1.3) | 0.26 | 14.3 (1.9) | **0.02** | 13.4 (1.4) | |  |  |
| - Interventricular septum, mm | 2.2 (0.4) | **<0.001** | 2.2 (0.4) | **<0.001** | 2.3 (0.4) | 0.08 | 2.5 (0.5) | |  |  |
| - Left ventricular posterior wall, mm | 1.8 (0.4) | **<0.001** | 1.8 (0.4) | **<0.001** | 1.9 (0.3) | 0.48 | 2.1 (0.7) | |  |  |
| - Left ventricular mass, g | 5.7 (1.4) | **<0.001** | 5.7 (1.2) | **<0.001** | 6.9 (2.1) | 0.21 | 6.4 (1.4) | |  |  |
| Values are presented as mean (SD) or as n (%). All p-values are comparisons with the control group. Significant p-values are marked with bold. | | | | | | | |  |  |  |
